# Risk Factors for Higher Volume of Hemorrhage in Ruptured Anterior Circulation Intracranial Aneurysms

**DOI:** 10.3389/fsurg.2020.587790

**Published:** 2020-11-12

**Authors:** Xiaolong Ya, Chaoqi Zhang, Jichao Liu, Shuo Zhang, Qian Zhang, Shuo Wang, Yong Cao, Jizong Zhao

**Affiliations:** ^1^Department of Neurosurgery, Beijing Tiantan Hospital, Capital Medical University, Beijing, China; ^2^Beijing Tiantan Hospital, Capital Medical University, Beijing, China; ^3^National Clinical Research Center for Neurological Diseases, Beijing, China

**Keywords:** ruptured aneurysm, anterior circulation, volume of hemorrhage, 3Dslicer, dangerous level

## Abstract

**Purpose:** To explore the influencing factors of volume hemorrhage in ruptured anterior circulation aneurysms, so as to identify the characteristics of anterior circulation aneurysms with high volume of hemorrhage, and to provide advice for clinical diagnosis and treatment for those aneurysms.

**Methods:** We retrospectively reviewed 437 cases of ruptured anterior intracranial aneurysms in our center between the years 2012 and 2017. According to the inclusion criteria, a total of 100 qualified patients were screened out. We collected demographic characteristics, environmental exposure, and admission status of enrolled patients. In addition, morphological parameters and location of aneurysms were also included. The semiautomatic threshold method was used to measure the volume of hemorrhage. According to the results, the patients were divided into the group with high blood volume and low blood volume. Univariate and multivariate logistic regression analyses were used to discover the related factors affecting the bleeding volume.

**Results:** In univariable analysis, pulse pressure (*P* = 0.014) showed a significant difference at the *P* < 0.05 test level. In terms of aneurysm morphology, the irregular shape (*P* < 0.001), calcification (*P* = 0.001), and flow angle (*P* = 0.006) showed significant statistical differences between the two groups at the *P* < 0.01 level (*P* < 0.01). Multivariate logistic regression analysis showed that irregular shape (OR = 5.370 *P* = 0.002 < 0.05), large flow angle (OR = 1.033 *P* = 0.016 < 0.05), and calcification (OR = 5.460 *P* = 0.003 < 0.05) were risk factors for volume of hemorrhage in ruptured anterior circulation aneurysms. The influence of hypertension history was at critical state (OR = 2.877 *P* = 0.051 > 005).

**Conclusions:** According to our analysis results, intracranial anterior circulation aneurysms with irregular shapes, calcifications, and large flow angle are more dangerous. Aneurysms with these characteristics often have a large amount of hemorrhage, requiring timely treatment in clinical practice.

## Introduction

Ruptured intracranial aneurysm is a severe disease of the central nervous system and usually results in a high rate of disability and mortality (1). Clinical practice and many studies have shown that the prognosis of ruptured intracranial aneurysms is mainly determined by the volume of the hemorrhage (2–5). Previous studies have quantified the amount of subarachnoid hemorrhage according to the division of cistern; however, they lack consistent conclusions (3, 6, 7). Normally, ruptured intracranial aneurysm could not only lead to subarachnoid hemorrhage but also cause intracerebral hematoma and epidural hematoma. However, the pattern of hemorrhage, in fact, may be more complex, and the mixed hemorrhage pattern may be more common, such as intracerebral hematoma with subarachnoid hemorrhage (8). Although there are a variety of quantitative scoring methods for cerebral hemorrhage, these methods are limited to a single hemorrhagic model, which leads to the imprecise assessment of aneurysm bleeding volume and development of the method that has the potential to be universally applicable to quantitative methods (3, 6, 9, 10).

In this study, we used the method of 3Dslicer semi-automatic calibration to quantify the volume of hemorrhage and explore the main risk factors. The aim was to grade the risk of ruptured aneurysms to identify the characteristics with high volume.

## Patient and Method

### Study Population

**Figure 1:**
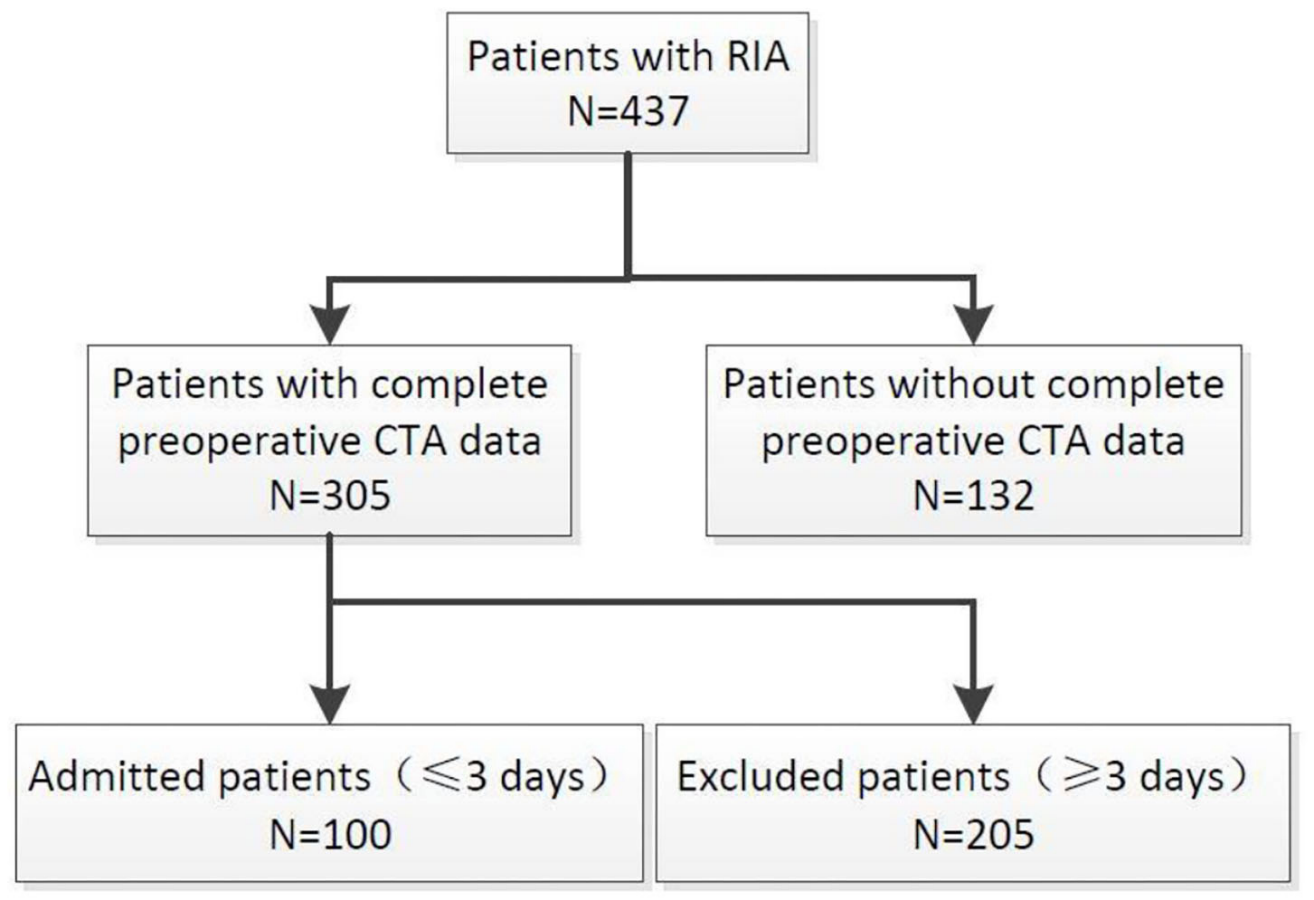
Flow diagram showing the selection of patients in this study. RIA, rupture intracranial aneurysm; UIA, unrupture intracranial aneurysm.

We retrospectively reviewed a consecutive series of patients with aneurysm admitted in our department between December 2012 and December 2017. We selected patients according to the following criteria: (1) Patients with intracranial aneurysm who visited the hospital from December 2012 to December 2017; (2) Patients with complete preoperative imaging data (including CT data of confirmed rupture and CTA original data of confirmed intracranial aneurysm); (3) Confirmed intracranial hemorrhage caused by aneurysm rupture; (4) Responsible aneurysm is located in the anterior circulation; (5) Patients with primary aneurysm rupture; and (6) Patients whose time from symptom onset to imaging diagnosis of intracranial hemorrhage was less than 3 days. Exclusion criteria are as follows: (1) Patients with unruptured intracranial aneurysm; (2). Aneurysms were diagnosed in patients with incomplete or absent imaging data (CT/CTA) in other hospitals and preoperative imaging data in our hospital; (3) Intracranial hemorrhage was determined to be caused by trauma, hypertension, cerebral amyloidosis, or other causes; (4) Responsible aneurysm is located in the posterior circulation; and (5) The time from onset to diagnosis of intracranial hemorrhage by CT imaging is more than 3 days. 305 patients had complete imaging data that mainly include CT data used for diagnosed cerebral hemorrhage and original CTA data used for diagnosed intracranial aneurysm. One hundred patients meet the inclusion criteria and were enrolled in the study (Figure 1).

### Acquisition of Variables

**Figure 2:**
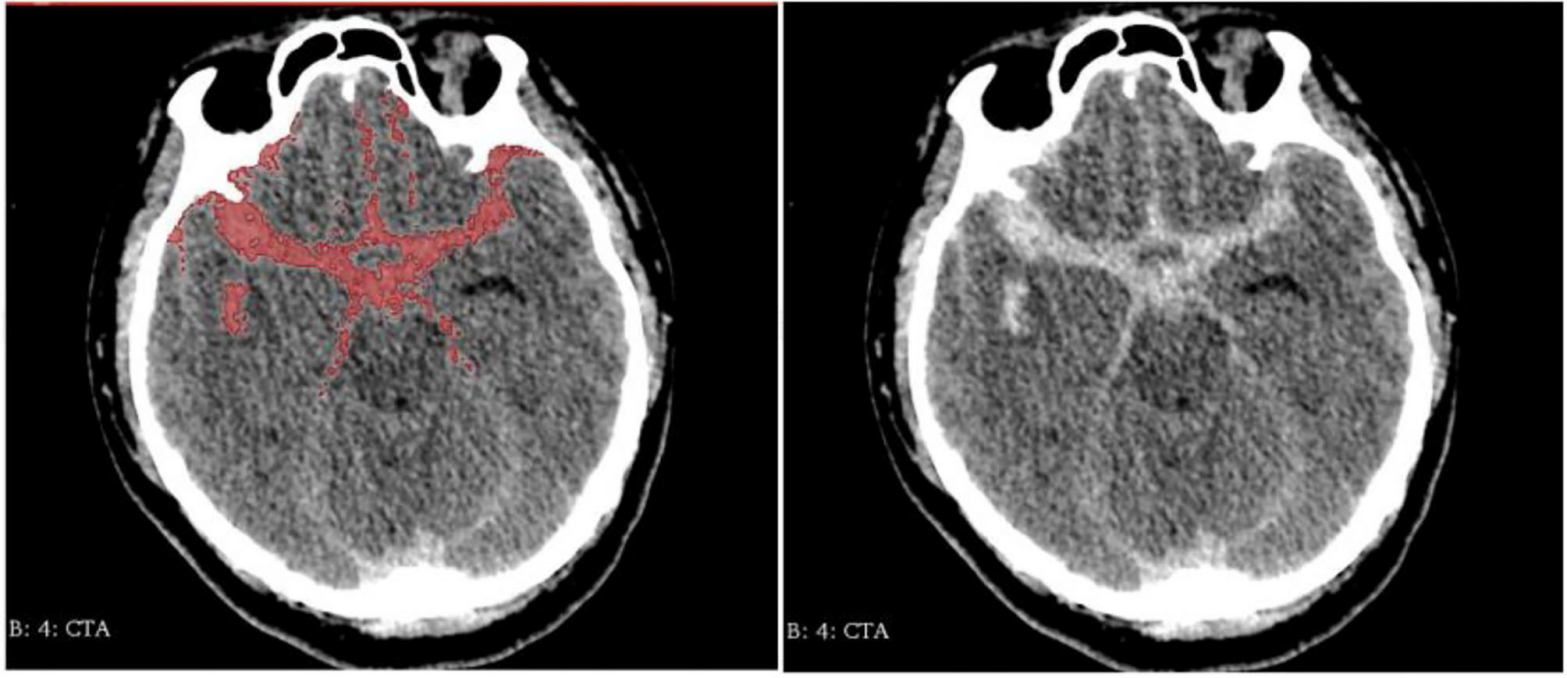
Region of interest be recognized by 3Dslicer (Left: identified area Right: original image).

**Figure 3:**
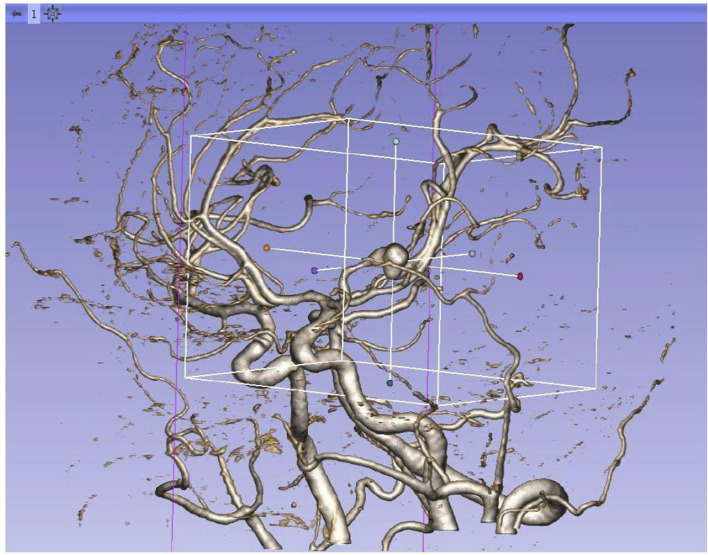
Reconstructed 3D CTA by 3Dslicer platform.

We reviewed clinical records and radiological data of the enrolled patients. Clinical variables include demographic data and previous history. Radiological variables include the volume of hemorrhage, location, and morphology parameters. Clinical variables were collected through the HIS system. Morphological parameters and location were collected by the 3Dslicer platform. The semiautomatic threshold method was used to measure the volume of hemorrhage. Thinner CT scans of enrolled patients were analyzed by using the 3Dslicer software platform (3Dslicer 4.11.0-2019-06-04 r28289). Regions of hemorrhage on CT scan were outlined slice by slice using a semiautomatic threshold approach (Figure 2: 3Dslicer recognize region of interest). All the hemorrhagic areas in different slices are added up automatically by the computer to obtain the final volume. Morphological data of aneurysms were measured using 3D CTA reconstructed from the 3Dslicer platform (Figure 3: 3D CTA). The radiological parameters were measured by five radiologists who were professionally trained in software operations. This study was approved by the Institutional Review Board of Beijing Tiantan Hospital, Capital Medical University. Written consents were obtained from the participant at the time of admission for research use.

### Statistical Analysis

Statistical analysis was performed by using SPSS Version 25.0. For statistical analysis, the volume of hemorrhage was dichotomized into the high group or low group based on the mean data. First, we used univariable analysis to discover the risk factors responsible for the volume of hemorrhage. For the analysis of continuous variables, data were evaluated whether it conforms to a normal distribution. If it was consistent, we use the *t*-test for analysis; otherwise, the nonparametric rank-sum test was adopted. For the analysis of categorical variables, if the number of spaces in the classification table is <5, Fisher's exact probability was used; otherwise, we adopted the Pearson chi-square test. Secondly, we included dominant factors, critical factors, and potential factors into the multivariate logistic regression model. Factors were considered to have a significant association with the volume of hemorrhage when the individual *P* value was <0.05 in a logistic regression model.

## Result

Among the 100 patients included in the study, 45 (45%) were male and 55 (55%) were female, with a male–female ratio of 1:1.2. The average age of the population is 51.6 ± 12.2 years old. Hypertension was diagnosed in 47% patients; a history of diabetes mellitus was found in 9% patients; and a history of cigarette smoking was found in 17% patients. Three percentage enrolled patients had a history of using anticoagulants (anticoagulants include antiplatelet drugs and anticoagulants). The mean time from symptom onset to hemorrhage diagnosed by CT scan was 37.5 ± 23.1 h. The preadmission systolic blood pressure (SBP) was 148.3 ± 18.0 mmHg.

**Figure 4:**
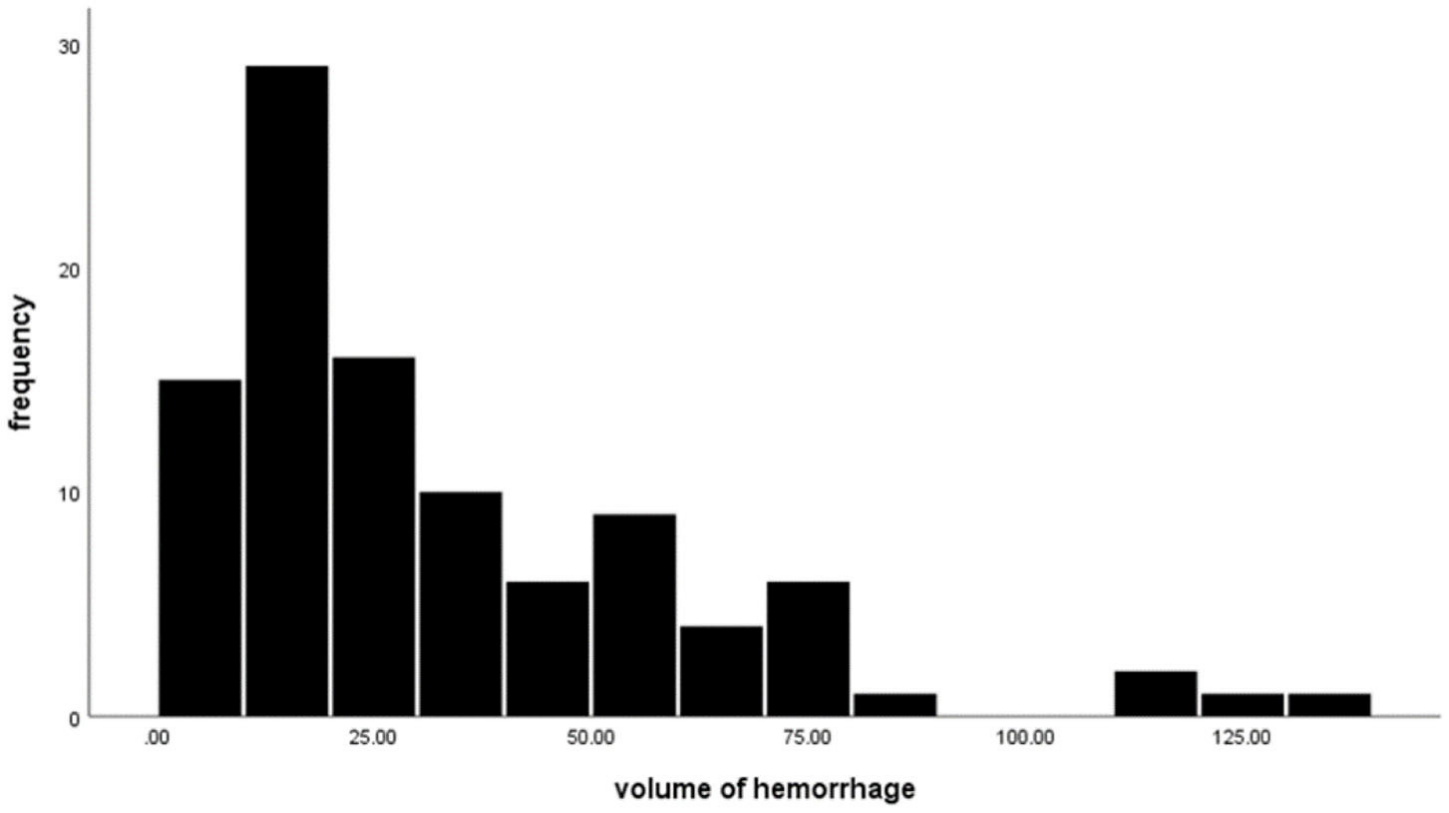
Frequency distribution of volume of hemorrhage.

The location of aneurysms in enrolled patients is shown below: nine anterior cerebral artery (ACA) aneurysms, 38 middle cerebral artery (MCA) aneurysms, 13 internal carotid artery (ICA) aneurysms, 28 anterior communicating artery (ACOA) aneurysms, and 12 posterior communicating artery (PCOA) aneurysms. The aneurysmal size was 4.6 ± 2.9 mm [mean ± standard deviation (SD)], ranging from 0.8 to 15.1 mm. The aneurysmal neck length was 2.8 ± 1.5 mm, ranging from 0.6 to 12.2 mm. The flow angle of aneurysms was 124.6 ± 18.9, with the minimal and maximal values being 90 and 170. About 54% of aneurysms are irregularly shaped and 46% are regularly shaped. Among the enrolled patients, 17 had multiple aneurysms and 83 had one single aneurysm. The calcification rate of aneurysms was 27%, and 73 aneurysms showed no calcification. The average volume of hemorrhage was 33.6 ± 28 ml, ranging from 3.4 to 134.8 ml. Table 1 summarizes detailed information on patient characteristics. Figure 4 shows the distribution of hematoma.

**Table 1:** Patient characteristics.

**Parameter**	***n* or Mean (±SD)**	**Variables**	***n* or Mean (±SD)**
Gender		Aneurysm site	
Female	45	ICA	13
Male	55	ACoA	28
Age	51.6 ± 12.2	PCoA	12
Hypertension		ACA	9
Yes	53	MCA	38
No	47	Aneurysm size (mm)	4.6 ± 2.9
Diabetes		Neck length (mm)	2.8 ± 1.5
Yes	9	Aspect ratio^*^	1.8 ± 1.1
No	91	Flow angle	124.6 ± 18.9
Taking anticoagulant drugs		Shape	
Yes	22	Regular	46
No	78	Irregular	54
Time from onset to diagnosed (h)	37.5 ± 23.1	Calcification	
Preadmission SBP (mmHg)	148.3 ± 18.0	Yes	27
Volume of hemorrhage	33.6 ± 28	No	73
Smoking		Aneurysm number	
Yes	17	1	83
No	83	>1	17

* *Maximal distance from neck to aneurysm dome/maximal neck width*.

### Univariable Analysis

In univariate analysis, clinical and imaging variables were selected as independent variables. The bleeding volume was divided into the high group and the low group according to the mean value. In terms of clinical variables analysis, our results show that patients with a history of using anticoagulant drugs were related to a high volume of hemorrhage (*p* = 0.044 < 0.05). Besides, there were significant statistical differences between high group and low group in SBP (*P* = 0.028 < 0.05). In respect to radiological variable analysis, we identified that calcified aneurysm was significantly associated with the high volume of hemorrhage (*P* = 0.001 < 0.05). Aneurysms with large flow angle and irregular characteristics would lead to a high volume of hemorrhage (*P* = 0.006, *P* < 0.001). Univariable analysis results are shown in Table 2.

**Table 2:** Univariable analysis of risk factors of hemorrhage.

**Parameter**	**Low blood volume**	**High blood volume**	***p***
	***n*% or Mean (±SD)**	***n*% or Mean (±SD)**	
Male	27%	18%	0.451
Age	50.3 ± 12.4	54.0 ± 11.8	0.152
Smoking	13%	9%	0.587
Hypertension	28%	19%	0.385
Diabetes	4%	5%	0.277
Taking anticoagulant drugs	0%	3%	0.044^*^
Time-to-treat (h)	37.6 ± 23.6	37.5 ± 23.0	0.992
Preadmission SBP (mmHg)	145.4 ± 16.8	153.6 ± 19.0	0.028^*^
Aneurysm site			0.262
ICA	10	3	
ACoA	20	8	
PCoA	8	4	
ACA	7	2	
MCA	19	19	
Aneurysm size (mm)	4.7 ± 3.0	4.6 ± 2.6	0.900
Neck length (mm)	2.7 ± 1.7	2.7 ± 1.1	0.570
Aspect ratio	1.8 ± 0.9	1.8 ± 1.2	0.936
Flow angle	120.7 ± 17.6	131.4 ± 18.8	0.006^*^
Regular/Irregular	38/26	8/28	<0.001^*^
Calcification	10%	17%	0.001^*^
Aneurysm number (>1)	10%	7%	0.626

* *P values were statistically different*.

### Multivariable Analysis

The statistically significant risk factors from the univariate analysis were subsequently evaluated with a multivariate logistic regression analysis. Our data shows that large flow angle was significantly associated with high volume (OR = 1.033, 95% CI: 1.006–1.062, *p* = 0.016). Additionally, we identified that the irregular shape of aneurysms was a significant risk factor of high volume (OR = 5.370, 95% CI: 1.901–15.171, *p* = 0.002). Calcification is still associated with high blood volume (OR = 5.460, 95% CI: 1.757–16.965, *p* = 0.003). Although a high preadmission SBP was significantly related to high blood volume (*P* = 0.028); however, it was not a clinically relevant risk factor (OR = 1.039, *p* = 0.061). For the other variates, blood volume also did not correlate with them. The results of multivariate regression analysis are shown in Table 3.

**Table 3:** Multivariable analysis of risk factors of hemorrhage.

**Parameter**	**OR**	**95% CI**	***p***
Time-to-treat (h)	1.004	0.981–1.027	0.756
Hypertension	2.877	0.996–8.311	0.065
Diabetes	0.509	0.097–2.663	0.424
Preadmission SBP (mmHg)	1.039	0.998–1.082	0.061
Regular/Irregular	5.370	1.901–15.171	0.002^*^
Flow angle	1.033	1.006–1.062	0.016^*^
Calcification	5.460	1.757–16.965	0.003^*^
Aneurysm size (mm)	1.017	0.877–1.178	0.827
Aspect ratio	0.885	0.512–1.527	0.660

* *P values were statistically different*.

## Discussion

The severity of ruptured intracranial aneurysms varies widely in clinical observation. Some ruptured aneurysms have non-conspicuous CT appearance and mild symptoms, while others lead to fatal bleeding and even coma or death. Many studies have suggested that the amount of hemorrhage after aneurysmal rupture is closely related to the clinical symptoms and prognosis (4, 5, 11, 12). Although there are many methods to calculate the amount of subarachnoid hemorrhage and some of them also take into account the amount of intraventricular, it is also difficult to precisely measure the amount of bleeding due to the cumbersome assessment and observer error (12). In this study, we use the method of semiautomatic threshold measurement in 3Dslicer to automatically circle the area of bleeding and get a whole volume.

The finding of the current study shows that the amount of aneurysmal bleeding was independently related to shape, flow angle, and calcification. Many studies have shown that aneurysmal morphology-related factors are strong predictors of rupture risk (13, 14). In general, the irregular lumen may cause blood flow to swirl locally, making a lower wall shear stress (WSS) and higher oscillatory shear index (OSI), and thus increase the risk of aneurysm rupture (15). Zhang and Abboud, studying the correlation between geometric features of the aneurysms and volume of aneurysmal hemorrhage, reported that patients with irregular aneurysms have a high volume (10, 16). Our data also indicate that irregular aneurysms are an independent risk factor for the volume of aneurysmal hemorrhage (*P* = 0.003 < 0.01). Irregular morphology is a risk factor of both rupture and blood loss. The flow angle of aneurysms is also a common parameter to describe aneurysm morphology. Flow angle also has been reported by several studies as a risk factor for aneurysm rupture (17, 18). Compared with the small flow angle, aneurysms with large flow angle may have a higher bleeding volume after rupture (*P* = 0.014 < 0.05). We believe that aneurysms with a large flow angle may be more likely to receive a direct impact from blood and thus cause more bleeding during rupture. Surprisingly, there was no statistical correlation between size and volume. The conventional opinion is that, the larger the aneurysm, the greater will be the bleeding (10). Russell used the Hijdra score to measure the volume of SAH and showed that smaller aneurysms tend to cause higher hemorrhage volumes (7). However, Salary suggested that there was no correlation between the hemorrhage volume and the size of the aneurysm according to their study (6). Our results support Salary's opinion. This controversy about the relationship between size and volume existed in the past decades. Regarding the size as a crude morphological description, there are still some inevitable confounding factors. In addition, the lack of standards for size is also an essential reason for variable results. We hope to use a standard measuring method with a larger sample in the near future for a more in-depth analysis. In terms of aneurysm morphology, we suggest that intracranial aneurysms with these characters in anterior circulation should be actively managed by medical intervention.

Interestingly, we found that calcified aneurysms were strongly associated with more blood loss (*P* = 0.001 < 0.05). It is generally believed that the walls of calcified aneurysms will be stronger and less likely to rupture, but some scholars believe calcified aneurysms may grow in size with time. Calcification in the artery wall can initiate local chronic inflammation, stimulating tissue and leading to local ulcers, which lead to the development of aneurysms (19, 20). Although some scholars believe that artery wall calcification will make aneurysm stronger, we find that calcification was usually confined to a certain part of the wall, which indicates the enhancement of the local stiffness. Some studies also have shown that calcification in some aneurysms increases its rigidity and decreases its elasticity (21). The presence of calcified plaque can decline the compliance of the entire cystic wall. Once the aneurysm ruptures, the cystic wall cannot rely on the shrinkage effect to reduce the size of the cavity and lead to higher blood loss. Although calcification can make surgery more complicated, it is important to recommend that those unruptured calcified aneurysms should be treated to prevent the fatal consequences after rupture.

This study had some limitations. First, the study was retrospectively designed. It is unavoidable that there is potential selection bias and confounders could not be totally excluded. Second, the sample size of this study was relatively small; for example, there are only three enrolled patients taking anticoagulant drugs. Considering the reason of the experimental error, only single-factor correlation analysis has to be performed. Future studies are required to confirm this effect between coagulation function and blood volume. Finally, all of the enrolled patients were with anterior circulation intracranial aneurysms; the results showed low ductility, which could not sufficiently explain the conditions of other aneurysms.

## Conclusion

Intracranial anterior circulation aneurysms with irregular shapes, calcifications, and large flow angle are more dangerous. Aneurysms with these characteristics tend to have greater blood volume and require active treatment in clinical practice.

## Data Availability Statement

The raw data supporting the conclusions of this article will be made available by the authors, without undue reservation.

## Ethics Statement

The studies involving human participants were reviewed and approved by Institutional Review Board of Beijing Tiantan Hospital, Capital Medical University. The patients/participants provided their written informed consent to participate in this study.

## Author Contributions

XY and QZ: conception and design. XY, CZ, and JL: acquisition of data. XY: analysis and interpretation of data and drafting the article. SW, YC, QZ, and JZ: technical supports and surgery. JZ: study supervision. All authors supervised and approved the final version of the manuscript.

## Conflict of Interest

The authors declare that the research was conducted in the absence of any commercial or financial relationships that could be construed as a potential conflict of interest.
